# Pregnant women's nutrition knowledge levels and some blood parameters in gynecology and obstetrics clinic

**DOI:** 10.1590/1980-220X-REEUSP-2024-0439en

**Published:** 2025-05-09

**Authors:** Muradiye Karasu Ayata, Aybüke Tayarer, Halime Aydemir

**Affiliations:** 1 Kirsehir Ahi Evran University, Faculty of Health Sciences, Department of Nutrition and Dietetics, Kirsehir, Turkey.; 2 Kirsehir Ahi Evran University, Faculty of Medicine, Department of Obstetrics and Gynecology, Kirsehir, Turkey.; 3 Kirsehir Ahi Evran University, Faculty of Health Sciences, Department of Midwifery, Kirsehir, Turkey.

**Keywords:** Diet, Healthy, Blood, Nutrition, Pregnancy, Dieta Saudável, Sangue, Nutrição, Gravidez

## Abstract

**Objective:**

To determine the nutritional knowledge and blood parameters in pregnant women.

**Method:**

This cross-sectional study included 108 pregnant women who attended the gynecology and obstetrics clinic in Kirsehir Province, Turkey. Sociodemographic information was gathered, as well as the habits of the pregnant women, information related to obstetrics and nutrition education, and the average score on the nutrition knowledge level scale. In addition, the average values of several blood parameters were determined.

**Results:**

The average Nutrition Knowledge Level Scale (NKLS) scores for adults and pregnant women were above the midpoint of the scale. The average values of the blood parameters for these women are as follows: hemoglobin 12,35 ± 1,32, hematocrit 36,81 ± 3,34, protein 66,93 ± 5,33, folic acid 14,62 ± 5,21, calcium 8,85 ± 0,62, creatinine 0,52 ± 0,08, and albumin 3,84 ± 0,48. The proportion of pregnant women with hemoglobin levels < 11 g/dl was 12,9%.

**Conclusion:**

This was the first study to examine the nutritional status of pregnant women in this province. This shows the importance of informing pregnant women about nutrition from the preconception period onwards.

## RESUMO

**Objetivo:**

Determinar os níveis de conhecimento sobre nutrição e alguns parâmetros sanguíneos em mulheres grávidas.

**Método:**

Este estudo transversal incluiu 108 mulheres grávidas que solicitaram atendimento em uma clínica de ginecologia e obstetrícia na província de Kirsehir, Turquia. Foram coletadas informações sociodemográficas, hábitos das gestantes, dados relacionados à obstetrícia e à educação nutricional, além da pontuação média da escala de nível de conhecimento nutricional. Também foram determinadas as médias de alguns parâmetros sanguíneos.

**Resultados:**

As pontuações médias da Escala de Nível de Conhecimento Nutricional para Adultos das gestantes foram superiores à metade da pontuação média da escala. Os valores médios dos parâmetros sanguíneos dessas mulheres foram os seguintes: hemoglobina 12,35 ± 1,32, hematócrito 36,81 ± 3,34, proteína 66,93 ± 5,33, ácido fólico 14,62 ± 5,21, cálcio 8,85 ± 0,62, creatinina 0,52 ± 0,08 e albumina 3,84 ± 0,48. A proporção de mulheres grávidas com níveis de hemoglobina abaixo de 11 g/dL foi de 12,9%.

**Conclusão:**

Este é o primeiro estudo sobre nutrição em gestantes realizado nesta província. Os resultados destacam a importância de fornecer informações sobre nutrição às mulheres grávidas desde o período pré-concepcional.

## INTRODUCTION

Nutrition involves the utilization of nutrients for growth, development, and overall health maintenance. Inadequate or unbalanced nutrition increases the risk of decreased immunity and illness, particularly in pregnant and lactating women^(1)^.

Adequate and balanced nutrition is one of the most important factors affecting the health of mothers and fetuses during pregnancy. Many studies have demonstrated a relationship between nutrition during pregnancy and birth weight, brain development, intrauterine death, premature birth, and preeclampsia. Failure to meet increased nutrient and energy requirements during pregnancy may result in maternal weight loss or the development of certain diseases.

The nutritional needs of pregnant women should be determined by considering factors such as age, physical activity status, pre-pregnancy weight, and nutrient stores^(2)^. If these increased nutritional needs are not met during pregnancy, conditions such as anemia and other health issues may arise. Inadequate and unbalanced nutrition in women is associated with a higher incidence of toxemia. Pregnant women with inadequate nutrition are more likely to develop megaloblastic anemia due to folate and vitamin B12 deficiencies^(3)^.

During pregnancy, adequate levels of macro-and micronutrients are consumed, which are crucial for the healthy development of the fetus. Vitamin D deficiency during pregnancy can lead to rickets, delayed bone mineralization, and hypocalcemia in the child^(4)^. Inadequate folate, which plays a role in nucleic acid synthesis, can lead to neural tube defects, premature birth, increased risk of low birth weight, and intrauterine growth restriction^(5)^. Iron deficiency anemia during pregnancy also increases the risk of premature birth and low birth weight^(6)^. In Turkey, a high proportion of women of childbearing age and children, significant infant and child mortality rates, and inadequate and low-quality nutrition education across all age groups are the main factors negatively affecting maternal and child health. Accessing Body Mass Index (BMI) and regulating weight gain are essential aspects of prenatal care. Raising awareness among healthcare professionals, evaluating maternal nutrition during prenatal visits, and providing necessary guidance are crucial for ensuring a healthy future^(7)^.

A healthy pregnancy results in changes in blood composition and biochemical parameters. While total iron-binding capacity, iron, calcium, magnesium, and hemoglobin decrease, the levels of triglycerides, leukocytes, HDL, LDL, and total cholesterol increase. An abnormal or decrease in these parameters may raise the risk of complications such as hypertension, anemia, premature birth, and low birth weight. Therefore, regular examination, monitoring, and prevention of deficiencies in these parameters from pre-pregnancy to pregnancy are crucial for a healthy pregnancy and normal fetal development^(8)^. Studies indicate that excess folic acid can trigger carcinogenicity, negatively affect zinc absorption, lead to delays in diagnosis and intervention due to vitamin B12 deficiency, and is associated with risks of asthma, allergies, and autism. Additionally, high vitamin D intake is associated with hypercalcemia^(9)^.

Nutritional knowledge and literacy are crucial for healthy eating, improving diet quality, and the healthy termination of pregnancy. Pregnant women with better nutritional knowledge can adjust their eating habits to improve their health, that of their families, and the community^(10)^. This study aimed to determine the level of nutritional knowledge and blood parameters of pregnant women attending a gynecology and obstetrics clinic.

## METHOD

### Study Design and Setting

This is a cross-sectional study. Research data were collected from pregnant women admitted to the Obstetrics and Gynecology Outpatient Clinic of the Kirsehir Ahi Evran University Training and Research Hospital between March 1 and June 30, 2023.

### Study Population and Sample

The study population consisted of pregnant women who visited the Obstetrics and Gynecology Outpatient Clinic of Kirsehir Ahi Evran University Training and Research Hospital in the 2nd and 3rd trimesters between March 1 and June 30, 2023. The study sample consisted of 108 pregnant women who met the inclusion criteria in their 2nd and 3rd trimesters between March 1 and June 30, 2023. The purpose and content of the research were explained to the pregnant women.

### Participants

The study included the voluntary participation of pregnant women who were citizens of the Republic of Turkey and in the 2nd or 3rd trimester (pregnant after the 12th week of gestation). Pregnant women who did not complete the survey were excluded. Face-to-face interviews were conducted with pregnant women who visited the Clinic and the Research Hospital for the survey form and scale questions. The routine hemogram and biochemistry parameter results in the prenatal check-ups of the pregnant women were obtained from the hospital information system and recorded in the Blood Parameters and Results Form.

### Data Collection Tools

#### Survey Form

The survey questionnaire, prepared in line with studies in the literature, consisted of 24 questions regarding sociodemographic characteristics, obstetric features, habits, receiving nutrition education, sources of nutritional information, daily water consumption, iron and folic acid intake status, and pregnancy complaints of pregnant women^(11,12,13,14,15)^. The Nutrition Knowledge Level Scale (NKLS) for Adults was used to assess the nutritional knowledge level of pregnant women. Permission to use the scale was obtained from the relevant authors.

#### NKLS for Adults

The NKLS for Adults, developed by Batmaz^(16)^ and validated for validity and reliability, consists of 20 items on “Basic nutrition and food-health relationship” and 12 items on “Food choice,” rated on a 5-point Likert scale. The response options were “strongly agree” (4 points), “agree” (3 points), “neither agree nor disagree” (2 points), “disagree” (1 point), and “strongly disagree” (0 points). The maximum scores are 80 for “Basic nutrition” and 48 for “Food choice”. The scale also includes separate Visual Analog Scale (VAS) assessments, ranging from 0 to 10, to evaluate participants’ perceptions of nutrition-health relationships and the accuracy of their food choices in daily life.

#### Hematological and Biochemical Parameters Form

This form contains the results of hematological and biochemical parameters. Routine hematological and biochemical blood results obtained during the examination and follow-up of pregnant women were obtained from the hospital’s patient information system. Blood samples were not collected from pregnant women.

### Ethical Aspects

Before the study, ethical approval was obtained from the Kirsehir Ahi Evran University Clinical Research Ethics Committee (decision n° 2023-03/22, February 7, 2023), and institutional permission was obtained from the Kirsehir Ahi Evran University Provincial Health Directorate Scientific Research Evaluation Committee (n° E-42884709, February 27, 2023).

### Statistical Analyses

The IBM Statistical Package for Social Sciences (SPSS) 21.0 program was used for the statistical analysis of the data. Descriptive statistical methods, including frequency distribution and arithmetic mean tests, were applied. The normal distribution of the data was confirmed by examining the Shapiro–Wilk and Kolmogorov-Smirnov tests for conformity to normal distribution, skewness, and kurtosis coefficients in each group, and graphical representations (histogram, normal Q-Q plot). The data followed a normal distribution. Independent-sample t-tests were conducted for independent groups. In the analysis, a p-value of less than 0.05 was considered statistically significant^(17,18)^.

In a study conducted to assess the validity and reliability, the internal consistency coefficient Cronbach’s Alpha was calculated to be 0.72 for the 20 items under the “Basic Nutrition” heading and 0.74 for the 12 items under the “Nutrition Choice” heading. In the present study, the internal consistency coefficient Cronbach’s Alpha was found to be 0.70 for the 20 items under the “Basic Nutrition” heading and 0.65 for the 12 items under the “Nutrition Choice” heading.

## RESULTS

The distribution of the sociodemographic characteristics and habits of the pregnant women is shown in Table 1.

**Table 1:** Distribution of socio-demographic and habits of pregnant women– Kirsehir, Turkey, 2024.

Variables	n	%
Mean age (Mean ± SD)	28.38 ± 5.35 (min. 18-max. 40)	
Average pre-pregnancy BMI (Mean ± SD)	25.49 ± 4.94 (min. 12.65-max. 41.33)	
Average during pregnancy BMI (Mean ± SD)	28.51 ± 4.84 (min. 17.21-max. 45.63)	
Pre-pregnancy BMI categories		
<18.5 18.5–24.9 25.0–29.9 30.0–34.9 35.0–39.9 >40	5 51 35 11 5 1	4.6 47.3 32.4 10.2 4.6 0.9
BMI categories during pregnancy		
<18.5 18.5–24.9 25.0–29.9 30.0–34.9 35.0–39.9 >40	2 26 42 28 9 1	1.9 24.1 38.9 25.9 8.3 0.9
Education level		
Illiterate Literate Primary school High school University Postgraduate	1 2 12 43 46 4	0.9 1.9 11.1 39.8 42.6 3.7
Employment status		
Employed	38	35.19
Unemployed (Housewife)	70	64.81
Income level		
Low Moderate High	12 84 12	11,1 77.8 11.1
Having a health problem		
No problems Having problems	93 15	86.1 13.9
Smoking status		
Not smoking Smoking	97 11	89.8 10.2
Drinking alcohol		
Not drinking Drinking	108 0	100.0 0.0
Average daily water consumption (Mean ± SD)	1745.37 ± 888.07 (min. 400-max. 5000)	
Total	108	100.0

Table 2 shows the distribution of obstetric information for the pregnant women. About half were in the second and third trimester based on gestational weeks (Table 2).

**Table 2:** Distribution of obstetric information of pregnant women – Kirsehir, Turkey, 2024.

Variables	n	%
Trimester status by gestational week		
2nd Trimester	57	52.8
3rd Trimester	51	47.2
Average number of pregnancies (Mean ± SD)	2.23 ± 1.14 (min. 1 − max. 5)	
Average number of births (Mean ± SD)	1.00 ± 0.99 (min. 0 − max. 4)	
Status of complaints during pregnancy		
No complaints Experiencing complaints	43 65	39.8 60.2
Complaints experienced during pregnancy*		
Nausea	24	22.2
Vomiting	13	12.0
Constipation	12	11.1
Heartburn	44	40.7
Reflux	16	14.8
Indigestion	12	11.1
Other	13	12.0
Having an OGTT status (in pregnancies greater than 28 weeks 0 days)		
Didn’t have it done Had it done	25 24	51.1 48.9
Iron intake status during pregnancy		
Not taking Taking	31 77	28.7 71.3
Folic acid intake status during pregnancy		
Not taking Taking	35 73	32.4 67.6
Total	108	100.0

* Multiple options are included.

Table 3 presents the distribution of nutrition education among pregnant women. A total of 22.2% had received nutrition education, with the highest rate (15.7%) from healthcare professionals or dietitians. Among pregnant women, 36.1% expressed interest in receiving healthy eating education and being invited to such sessions (Table 3). The average scores (Mean ± SD) were 56.61 ± 7.48 for basic nutrition, 35.45 ± 5.22 for food choice, 8.16 ± 2.07 for the perceived relationship between nutrition and health, and 6.16 ± 1.93 for the perceived accuracy of their food choices in daily life.

**Table 3:** Distribution of information on nutrition education of pregnant women – Kirsehir, Turkey, 2024.

Variables	n	%
Receiving healthy nutrition education		
Uneducated Educated	84 24	77.8 22.2
Where to get healthy nutrition education?*		
Newspaper/magazine School Parents, environment Health personnel or dietitian Television/radio/social media Other	7 4 4 17 3 1	6.5 3.7 3.7 15.7 2.8 0.9
Wanting to receive healthy nutrition education		
Doesn’t want Wants	69 39	63.9 36.1
A desire to be invited to healthy nutrition training		
Doesn’t want Wants	69 39	63.9 36.1
Total	108	100.0

* Multiple options are included.

Table 4 compares the average scale scores based on the status of nutritional education among the pregnant women. An independent t-test was used to assess differences in Basic Nutrition and Food Choice scores between those who received healthy nutrition education and those who did not. The analysis revealed no significant differences (p > 0.05) in average scores (Table 4).

**Table 4:** Comparison of scale average scores according to nutrition education receiving status – Kirsehir, Turkey, 2024.

Healthy nutrition education receiving status	n	Mean ± SD	t*	p
Basic Nutrition				
Educated Not educated	24 84	57.79 ± 8.01 56.27 ± 7.34	0.833	0.411
Food Preference				
Educated Not educated	24 84	35.95 ± 4.62 35.30 ± 5.39	0.584	0.563

* Independent t-test (t).

Table 5 provides the distribution of the mean (Mean ± SD) of some blood parameters of pregnant women. The average blood parameter values for the pregnant women are shown in Table 5. The proportion of pregnant women with hemoglobin levels < 11 g/dl was 12.9%.

**Table 5:** Distribution of some blood parameter averages of pregnant women – Kirsehir, Turkey, 2024.

Averages of blood parameters	
Hemoglobin (Mean ± SD)	12.35 ± 1.32 (min. 6.80-max. 15.40) <11 g/dl = 12.9%
Hematocrit (Mean ± SD)	36.81 ± 3.34 (min. 24.70-max. 45.50)
Platelet (Mean ± SD)	249.18 ± 61.23 (min. 74-max. 459)
Protein (Mean ± SD)	66.93 ± 5.33 (min. 55-max. 86)
Folic acid (Mean ± SD)	14.62 ± 5.21 (min. 3-max. >20)
Calcium (Mean ± SD)	8.85 ± 0.62 (min. 6.60-max. 9.90)
Magnesium (Mean ± SD)	2.06 ± 0.47 (min. 1.10-max. 3.70)
Phosphorus (Mean ± SD)	3.28 ± 0.84 (min. 1.60-max. 9.10)
Iron (Mean ± SD)	64.12 ± 35.98 (min. 6.00-max. 171.00)
Zinc (Mean ± SD)	82.09 ± 16.60 (min. 46.32max. 150.00)
ALT (Mean ± SD)	12.74 ± 6.63 (min. 4.00-max. 46.00)
AST (Mean ± SD)	16.14 ± 5.56 (min. 8.00-max. 46.00)
HDL (Mean ± SD)	60.59 ± 14.33 (min. 34.00-max. 105.00)
LDL (Mean ± SD)	114.09 ± 40.96 (min. 10.50-max. 212.00)
Glucose (Mean ± SD)	88.22 ± 13.75 (min. 65.00-max. 134.00)
Triglyceride (Mean ± SD)	201.60 ± 90.20 (min. 57.00-max. 500.00)
TSH (Mean ± SD)	2.08 ± 2.34 (min. 0.01-max. 22.50)
T4 (Mean ± SD)	0.98 ± 0.23 (min. 0.05-max. 1.47)
Urea (Mean ± SD)	14.88 ± 3.91 (min. 6.00-max. 29.10)
Creatinine (Mean ± SD)	0.52 ± 0.08 (min. 0.34-max. 0.77)
Albumin (Mean ± SD)	3.84 ± 0.48 (min. 2.40-max. 5.17)

## DISCUSSION

The average age of the participants in this study was higher than that reported by Irge et al.^(7)^ and lower than the average age reported by Oymak^(3)^, while it was similar to the findings of Taraz^(10)^. Similarly, the BMI results indicated differences in the distribution of BMI categories compared with those in previous studies. The proportions of underweight, normal weight, pre-obese, and obese pregnant women in this study differed from those reported by Irge et al.^(7)^. Additionally, the prevalence of mild obesity was higher. These differences may be attributed to variations in the dietary habits specific to the regions in which the studies were conducted. Furthermore, a small percentage of the obese group was classified as morbidly obese.

It is important to note that maternal obesity can lead to numerous complications during pregnancy and postpartum and increase the risk of neonatal morbidity^(19)^. The high prevalence of obesity and its associated risks in this study highlights the need to address maternal obesity and its potential impact on maternal and neonatal health.

Based on a comparison with previous studies, this study showed notable differences in the educational levels and occupational status of pregnant women. The proportion of pregnant women with lower educational levels, such as primary school graduates or illiterate individuals, was significantly lower in this study than in the study by Irge et al.^(7)^. In contrast, the proportion of pregnant women with university-level education or above was lower, whereas the proportion of individuals with secondary school education or lower was higher, compared to the findings of Oymak^(3)^. These variations may be attributed to regional differences in educational attainment across different areas of Turkey.

Moreover, the percentage of pregnant women who were homemakers was lower than that reported by Irge et al.^(7)^ and Akaç^(11)^. This finding suggests a potential shift in the occupational status of pregnant women over time, which may have been influenced by societal and economic changes. In terms of income disparities, the present study reported a higher proportion of pregnant women with a household income matching their expenses compared to the findings of Tandoğan et al.^(20)^. These discrepancies in income levels highlight the potential impact of socioeconomic factors on the pregnant population and how these factors may vary across different regions. Overall, the comparison with previous studies revealed significant variations in the educational levels, occupational status, and income levels of pregnant women, which may reflect regional and temporal differences. These findings underscore the importance of considering socioeconomic factors in maternal and child health research and the need for targeted interventions to address potential disparities in access to education and income.

A comparison with the study by Irge et al.^(7)^ revealed variations in tobacco and alcohol consumption rates during pregnancy. In the study by Irge et al.^(7)^, the smoking rate during pregnancy was approximately half of that reported in this study, with no instances of alcohol consumption. These differences may be attributed to cultural factors that influence smoking and alcohol consumption during pregnancy.

Furthermore, when comparing daily water intake, the study found lower average daily water consumption among pregnant women compared to the findings of Taraz^(10)^. These differences highlight the potential influence of cultural and regional factors on maternal health behaviors and the importance of considering these factors in prenatal care and public health interventions.

The Ministry of Health’s iron supplementation program for pregnant women is an important step towards reducing the risk of iron deficiency anemia during pregnancy^(21)^. In the present study, the rate of iron supplementation intake was 71.3%, with a rate of 86.1% among pregnant women with at least a high school education. This finding suggests that a higher educational level may positively impact the frequency of iron supplementation.

Additionally, the average hemoglobin levels of pregnant women were 12.35 ± 1.32 g/dL, with an anemia prevalence of 12.9%. These findings indicate that most pregnant women who take iron supplements may balance this condition. Considering that World Health Organization^(22,23)^ data indicate an anemia prevalence of 40.2% among pregnant women in Turkey, the importance of iron supplementation is reaffirmed. Sunuwar et al. ^(24)^ reported that nutrition education was significantly associated with improved hemoglobin levels, dietary intake, and nutritional knowledge of anemia. Notably, 67.6% of the pregnant women in this study received folic acid supplementation, while 71.3% received iron supplementation. These rates may indicate the need for specific corrections in folic acid supplementation. These findings provide important information regarding the use of nutritional supplements and pregnancy-related complaints among pregnant women in Turkey.

Gestational diabetes mellitus (GDM) screening has been controversial in recent discussions. However, national and international organizations emphasize that performing an oral glucose tolerance test (OGTT) between the 24th and 28th weeks of gestation can reduce the complications associated with GDM and improve perinatal outcomes. According to Çakır and Yeşilçiçek Çalık^(25)^, the frequency of undergoing OGTT was determined to be 55.7%. In this study, 45.3% of the pregnant women were beyond 28 weeks of gestation from their last menstrual period, and 48.9% of them had undergone OGTT, similar to the findings of Çakır and Yeşilçiçek Çalık (2020)’s study^(25)^. The low frequency of the OGTT may be attributed to the negative impact of the median and poor health literacy.

Various complaints occur during pregnancy due to physical, mental, social, and anatomical factors related to the growing uterus. According to Karaçam and Özçelik^(26)^, 99.8% of pregnant women in Brazil complain during their pregnancies^(26)^. In this study, the rate of at least one complaint during pregnancy was 60.2%. This differing conclusion from that of Karaçam and Özçelik^(26)^ can be explained by the fact that only 47.2% of the included pregnant women were in their third trimester. In another study, the most common complaint among pregnant women was nausea (34.7%), followed by vomiting (10.9%)^(7)^. In the present study, the rates of nausea and vomiting were lower and higher, respectively.

In Önen and Güngör^(27)^ research, the mean basic nutrition score was reported to be 52 ± 5 in pregnant women in the second trimester and 52 ± 6 in the third trimester. In the present study, mean basic nutritional scores were higher. Önen and Güngör^(27)^ also reported an average food preference score of 38 ± 5 in their study, while the average food preference score was lower in the present study. These differences may be associated with the knowledge of nutrition and educational background. Although the average triglyceride and total cholesterol levels were lower than those reported by Brizzi et al.^(28)^, they were similar to those reported by Özdemirci et al.^(29)^.

In our study, while urea levels were low, which is consistent with the findings of Özdemirci et al.^(29)^, total protein, albumin, calcium, and hematocrit levels were within the normal reference ranges, in contrast to their results. Decreased urea levels are thought to be associated with an increased glomerular filtration rate, particularly during the third trimester. Although the average folate levels were within the laboratory reference ranges, they were higher in the study conducted by Comba and Mert^(30)^ (4.9 ng/ml). This difference can be explained by the smaller sample size in the previous study compared to the present study.

## CONCLUSION

This study is the first to examine the nutritional status of pregnant women in Kirsehir Province in Turkey. The average scores of pregnant women on the NKLS for Adults were higher than half the average scale score. While some of the blood parameters were within normal values, only the urea level was lower than normal.

Providing pregnant women with nutritional education is crucial. Nutrition education should start during the preconception period, and pregnant women should be regularly monitored and informed by knowledgeable dietitians and healthcare professionals.
