# Supplementary material for: Pregnant women's nutrition knowledge levels and some blood parameters in gynecology and obstetrics clinic
Source: Rev Esc Enferm USP. 2025 May 9;59:e20240439. doi: 10.1590/1980-220X-REEUSP-2024-0439en (PMC12068244; doi:10.1590/1980-220X-REEUSP-2024-0439en)
Supplement: Translation

# Translation: Niveles de conocimiento sobre nutrición de mujeres embarazadas y algunos parámetros sanguíneos en la clínica de ginecología y obstetricia

**DOI:** 10.1590/1980-220X-REEUSP-2024-0439es

**Authors:** Muradiye Karasu Ayata, Aybüke Tayarer, Halime Aydemir

**Affiliations:** 1 Kirsehir Ahi Evran University, Faculty of Health Sciences, Department of Nutrition and Dietetics, Kirsehir, Turkey.; 2 Kirsehir Ahi Evran University, Faculty of Medicine, Department of Obstetrics and Gynecology, Kirsehir, Turkey.; 3 Kirsehir Ahi Evran University, Faculty of Health Sciences, Department of Midwifery, Kirsehir, Turkey.

**Keywords:** Dieta Saludable, Sangre, Nutrición, Embarazo

## Abstract

**Objetivo:**

Determinar los conocimientos nutricionales y los parámetros sanguíneos en mujeres embarazadas.

**Método:**

Este estudio transversal incluyó a 108 mujeres embarazadas que acudieron a la clínica de ginecología y obstetricia de la provincia de Kirsehir, Turquía. Se recopiló información sociodemográfica, así como los hábitos de las mujeres embarazadas, información relacionada con la obstetricia y la educación nutricional, y la puntuación media en la escala de nivel de conocimientos nutricionales. Además, se determinaron los valores medios de varios parámetros sanguíneos.

**Resultados:**

Las puntuaciones medias de la Escala de Nivel de Conocimientos Nutricionales (NKLS) para adultos y mujeres embarazadas estaban por encima del punto medio de la escala. Los valores medios de los parámetros sanguíneos de estas mujeres son los siguientes: hemoglobina 12,35 ± 1,32, hematocrito 36,81 ± 3,34, proteínas 66,93 ± 5,33, ácido fólico 14,62 ± 5,21, calcio 8,85 ± 0,62, creatinina 0,52 ± 0,08 y albúmina 3,84 ± 0,48. La proporción de mujeres embarazadas con niveles de hemoglobina < 11 g/dl fue del 12,9 %.

**Conclusión:**

Este fue el primer estudio que examinó el estado nutricional de las mujeres embarazadas en esta provincia. Esto demuestra la importancia de informar a las mujeres embarazadas sobre la nutrición desde el período previo a la concepción en adelante.

## INTRODUCCIÓN

La nutrición implica la utilización de nutrientes para el crecimiento, el desarrollo y el mantenimiento general de la salud. Una nutrición inadecuada o desequilibrada aumenta el riesgo de disminución de la inmunidad y de enfermedad, especialmente en mujeres embarazadas y lactantes^(1)^.

Una nutrición adecuada y equilibrada es uno de los factores más importantes que afectan a la salud de las madres y los fetos durante el embarazo. Muchos estudios han demostrado una relación entre la nutrición durante el embarazo y el peso al nacer, el desarrollo cerebral, la muerte intrauterina, el parto prematuro y la preeclampsia. El incumplimiento de los mayores requerimientos de nutrientes y energía durante el embarazo puede resultar en pérdida de peso materno o en el desarrollo de ciertas enfermedades.

Las necesidades nutricionales de las mujeres embarazadas deben determinarse teniendo en cuenta factores como la edad, el estado de actividad física, el peso previo al embarazo y las reservas de nutrientes^(2)^. Si no se satisfacen estas mayores necesidades nutricionales durante el embarazo, pueden surgir afecciones como anemia y otros problemas de salud. La nutrición inadecuada y desequilibrada en las mujeres se asocia con una mayor incidencia de toxemia. Las mujeres embarazadas con una nutrición inadecuada son más propensas a desarrollar anemia megaloblástica debido a deficiencias de folato y vitamina B12^(3)^.

Durante el embarazo, se consumen niveles adecuados de macro y micronutrientes, que son cruciales para el desarrollo saludable del feto. La deficiencia de vitamina D durante el embarazo puede provocar raquitismo, retraso en la mineralización ósea e hipocalcemia en el niño^(4)^. El folato inadecuado, que desempeña un papel en la síntesis de ácidos nucleicos, puede provocar defectos del tubo neural, parto prematuro, mayor riesgo de bajo peso al nacer y restricción del crecimiento intrauterino^(5)^. La anemia ferropénica durante el embarazo también aumenta el riesgo de parto prematuro y bajo peso al nacer^(6)^. En Turquía, una alta proporción de mujeres en edad fértil y de niños, unas tasas de mortalidad infantil y juvenil significativas y una educación nutricional inadecuada y de baja calidad en todos los grupos de edad son los principales factores que afectan negativamente a la salud maternoinfantil. El acceso al índice de masa corporal (IMC) y la regulación del aumento de peso son aspectos esenciales de la atención prenatal. Sensibilizar a los profesionales sanitarios, evaluar la nutrición materna durante las visitas prenatales y proporcionar la orientación necesaria son cruciales para garantizar un futuro saludable^(7)^.

Un embarazo saludable da lugar a cambios en la composición de la sangre y en los parámetros bioquímicos. Mientras que la capacidad total de fijación del hierro, el hierro, el calcio, el magnesio y la hemoglobina disminuyen, los niveles de triglicéridos, leucocitos, HDL, LDL y colesterol total aumentan. Una anomalía o disminución de estos parámetros puede aumentar el riesgo de complicaciones como hipertensión, anemia, parto prematuro y bajo peso al nacer. Por lo tanto, el examen, el seguimiento y la prevención regulares de las deficiencias en estos parámetros desde el período previo al embarazo hasta el embarazo son cruciales para un embarazo saludable y un desarrollo fetal normal^(8)^. Los estudios indican que el exceso de ácido fólico puede desencadenar carcinogenicidad, afectar negativamente a la absorción de zinc, provocar retrasos en el diagnóstico y la intervención debido a la deficiencia de vitamina B12 y está asociado con riesgos de asma, alergias y autismo. Además, la ingesta elevada de vitamina D está asociada con la hipercalcemia^(9)^.

Los conocimientos y la alfabetización nutricionales son cruciales para una alimentación saludable, la mejora de la calidad de la dieta y la interrupción saludable del embarazo. Las mujeres embarazadas con mejores conocimientos nutricionales pueden ajustar sus hábitos alimentarios para mejorar su salud, la de sus familias y la de la comunidad^(10)^. Este estudio tenía como objetivo determinar el nivel de conocimientos nutricionales y los parámetros sanguíneos de las mujeres embarazadas que acuden a una clínica de ginecología y obstetricia.

## MÉTODO

### Diseño y Lugar del Estudio

Se trata de un estudio transversal. Los datos de la investigación se recopilaron de mujeres embarazadas ingresadas en la Clínica de Obstetricia y Ginecología del Hospital Universitario de Formación e Investigación de Kirsehir Ahi Evran entre el 1 de marzo y el 30 de junio de 2023.

### Población y Muestra del Estudio

La población del estudio consistió en mujeres embarazadas que visitaron la Clínica de Obstetricia y Ginecología del Hospital Universitario de Formación e Investigación Kirsehir Ahi Evran en el segundo y tercer trimestre entre el 1 de marzo y el 30 de junio de 2023. La muestra del estudio consistió en 108 mujeres embarazadas que cumplían los criterios de inclusión en su segundo y tercer trimestre entre el 1 de marzo y el 30 de junio de 2023. Se explicó a las mujeres embarazadas el propósito y el contenido de la investigación.

### Participantes

El estudio incluyó la participación voluntaria de mujeres embarazadas que eran ciudadanas de la República de Turquía y que se encontraban en el segundo o tercer trimestre (embarazadas después de la semana 12 de gestación). Se excluyeron las mujeres embarazadas que no completaron la encuesta. Se realizaron entrevistas cara a cara con las mujeres embarazadas que acudieron a la clínica y al hospital de investigación para rellenar el formulario de la encuesta y las preguntas de la escala. Los resultados de los parámetros bioquímicos y del hemograma rutinario de los controles prenatales de las mujeres embarazadas se obtuvieron del sistema de información del hospital y se registraron en el formulario de parámetros y resultados sanguíneos.

### Herramientas de Recopilación de Datos

#### Formulario de Encuesta

El cuestionario de la encuesta, elaborado de acuerdo con los estudios de la literatura, constaba de 24 preguntas sobre características sociodemográficas, características obstétricas, hábitos, educación nutricional recibida, fuentes de información nutricional, consumo diario de agua, estado de ingesta de hierro y ácido fólico, y quejas de embarazo de las mujeres embarazadas^(11–15)^. Se utilizó la Escala de Nivel de Conocimiento Nutricional (Nutrition Knowledge Level Scale - NKLS en la sigla en inglés) para Adultos para evaluar el nivel de conocimiento nutricional de las mujeres embarazadas. Se obtuvo permiso de los autores pertinentes para utilizar la escala.

#### NKLS Para Adultos

La NKLS para adultos, desarrollada por Batmaz^(16)^ y validada en cuanto a validez y fiabilidad, consta de 20 ítems sobre «Nutrición básica y relación entre alimentación y salud» y 12 ítems sobre «Elección de alimentos», calificados en una escala Likert de 5 puntos. Las opciones de respuesta eran «totalmente de acuerdo» (4 puntos), «de acuerdo» (3 puntos), «ni de acuerdo ni en desacuerdo» (2 puntos), «en desacuerdo» (1 punto) y «totalmente en desacuerdo» (0 puntos). Las puntuaciones máximas son 80 para «Nutrición básica» y 48 para «Elección de alimentos». La escala también incluye evaluaciones separadas de la Escala Visual Analógica (EVA), que van de 0 a 10, para evaluar las percepciones de los participantes sobre las relaciones entre nutrición y salud y la precisión de sus elecciones alimentarias en la vida diaria.

#### Formulario de Parámetros Hematológicos y Bioquímicos

Este formulario contiene los resultados de los parámetros hematológicos y bioquímicos. Los resultados hematológicos y bioquímicos rutinarios de sangre obtenidos durante el examen y seguimiento de las mujeres embarazadas se obtuvieron del sistema de información de pacientes del hospital. No se tomaron muestras de sangre de las mujeres embarazadas.

### Aspectos Éticos

Antes del estudio, se obtuvo la aprobación ética del Comité de Ética de Investigación Clínica de la Universidad de Kirsehir Ahi Evran (decisión n^.º^ 2023-03/22, 7 de febrero de 2023), y se obtuvo el permiso institucional del Comité de Evaluación de la Investigación Científica de la Dirección Provincial de Salud de la Universidad de Kirsehir Ahi Evran (n^.º^ E-42884709, 27 de febrero de 2023).

### Análisis Estadístico

Para el análisis estadístico de los datos se utilizó el programa IBM Statistical Package for Social Sciences (SPSS) 21,0. Se aplicaron métodos estadísticos descriptivos, incluidas pruebas de distribución de frecuencias y de media aritmética. La distribución normal de los datos se confirmó examinando las pruebas de Shapiro-Wilk y Kolmogorov-Smirnov para determinar la conformidad con la distribución normal, los coeficientes de asimetría y curtosis en cada grupo, y las representaciones gráficas (histograma, diagrama Q-Q normal). Los datos siguieron una distribución normal. Se realizaron pruebas t de muestras independientes para grupos independientes. En el análisis, un valor p inferior a 0,05 se consideró estadísticamente significativo^(17,18)^.

En un estudio realizado para evaluar la validez y fiabilidad, el coeficiente de consistencia interna alfa de Cronbach se calculó en 0,72 para los 20 ítems bajo el encabezado «Nutrición básica» y 0,74 para los 12 ítems bajo el encabezado «Elección de la nutrición». En el presente estudio, el coeficiente de consistencia interna alfa de Cronbach fue de 0,70 para los 20 ítems bajo el encabezado «Nutrición básica» y de 0,65 para los 12 ítems bajo el encabezado «Elección de la nutrición».

## RESULTADOS

La distribución de las características sociodemográficas y los hábitos de las mujeres embarazadas se muestra en la Tabla 1.

**Tabla 1:** Distribución sociodemográfica y hábitos de las mujeres embarazadas – Kirsehir, Turquía, 2024.

Variables	n	%
Edad media (Media ± DE)	28,38 ± 5,35 (mín. 18-máx. 40)	
IMC medio antes del embarazo (Media ± DE)	25,49 ± 4,94 (mín. 12,65-máx. 41,33)	
IMC medio durante el embarazo (Media ± DE)	28,51 ± 4,84 (mín. 17,21-máx. 45,63)	
Categorías de IMC antes del embarazo		
<18,5 18,5–24,9 25,0–29,9 30,0–34,9 35,0–39,9 >40	5 51 35 11 5 1	4,6 47,3 32,4 10,2 4,6 0,9
Categorías de IMC durante el embarazo		
<18,5 18,5–24,9 25,0–29,9 30,0–34,9 35,0–39,9 >40	2 26 42 28 9 1	1,9 24,1 38,9 25,9 8,3 0,9
Nivel de estudios		
Analfabetos Alfabetización Escuela primaria Bachillerato Universidad Postgrado	1 2 12 43 46 4	0,9 1,9 11,1 39,8 42,6 3,7
Situación laboral		
Empleado	38	35,19
Desempleada (ama de casa)	70	64,81
Nivel de ingresos		
Bajo Moderado Alta	12 84 12	11,1 77,8 11,1
Tener un problema de salud		
Sin problemas Problemas	93 15	86,1 13,9
Tabaquismo		
No fumar Fumar	97 11	89,8 10,2
Consumo de alcohol		
No beber Beber	108 0	100,0 0,0
Consumo medio diario de agua (Media ± DE)	1745,37 ± 888,07 (mín. 400-máx. 5000)	
Total	108	100,0

IMC: Índice de masa corporal; DE: Desvío Estándar.

La Tabla 2 muestra la distribución de la información obstétrica de las embarazadas. Aproximadamente la mitad se encontraban en el segundo y tercer trimestre según las semanas de gestación (Tabla 2).

**Tabla 2:** Distribución de la información obstétrica de las mujeres embarazadas – Kirsehir, Turquía, 2024.

Variables	n	%
Estado trimestral por semana gestacional		
2º trimestre	57	52,8
3er trimestre	51	47,2
Número medio de embarazos (Media ± DE)	2,23 ± 1,14 (mín. 1-máx. 5)	
Número medio de nacimientos (Media ± DE)	1,00 ± 0,99 (mín. 0-máx. 4)	
Estado de las quejas durante el embarazo		
Ninguna queja Reclamaciones	43 65	39,8 60,2
Molestias experimentadas durante el embarazo*.		
Náuseas	24	22,2
Vómitos	13	12,0
Estreñimiento	12	11,1
Acidez	44	40,7
Reflujo	16	14,8
Indigestión	12	11,1
Otros	13	12,0
Tener un estudio de tolerancia a glucosa (en embarazos de más de 28 semanas 0 días)		
No me lo hice Me lo hice	25 24	51,1 48,9
Consumo de hierro durante el embarazo		
No tomar En	31 77	28,7 71,3
Consumo de ácido fólico durante el embarazo		
No tomar En	35 73	32,4 67,6
Total	108	100,0

* Se incluyen múltiples opciones.

La Tabla 3 presenta la distribución de la educación nutricional entre las mujeres embarazadas. Un 22,2% había recibido educación nutricional, y la tasa más alta (15,7%) procedía de profesionales sanitarios o dietistas. Entre las embarazadas, el 36,1% manifestó su interés por recibir educación sobre alimentación saludable y por ser invitadas a sesiones de este tipo (Tabla 3). Las puntuaciones medias (Media ± DE) fueron 56,61 ± 7,48 para nutrición básica, 35,45 ± 5,22 para elección de alimentos, 8,16 ± 2,07 para la relación percibida entre nutrición y salud, y 6,16 ± 1,93 para la precisión percibida de sus elecciones alimentarias en la vida diaria.

**Tabla 3:** Distribución de la información sobre la educación nutricional de las mujeres embarazadas – Kirsehir, Turquía, 2024.

Variables	n	%
Recibir educación nutricional saludable		
Sin educación Educado	84 24	77,8 22,2
¿Dónde obtener información sobre nutrición saludable?*		
Periódico/revista Escuela Padres, medio ambiente Personal sanitario o dietista Televisión/radio/medios sociales Otros	7 4 4 17 3 1	6,5 3,7 3,7 15,7 2,8 0,9
Deseo de recibir educación nutricional saludable		
No quiere Quiere	69 39	63,9 36,1
Deseo de ser invitado a una formación sobre nutrición saludable		
No quiere Quiere	69 39	63,9 36,1
Total	108	100,0

* Se incluyen múltiples opciones.

En la Tabla 4 se comparan las puntuaciones medias de la escala en función del estado de la educación nutricional entre las embarazadas. Se utilizó una prueba t independiente para evaluar las diferencias en las puntuaciones de Nutrición Básica y Elección de Alimentos entre las que recibieron educación nutricional saludable y las que no. El análisis no reveló diferencias significativas (p > 0,05) en las puntuaciones medias (Tabla 4).

**Tabla 4:** Comparación de las puntuaciones medias de la escala según el estado de recepción de educación nutricional – Kirsehir, Turquía, 2024.

Estado de recepción de educación nutricional saludable	n	Media ± DE	t*	p
Nutrición básica				
Educado Sin estudios	24 84	57,79 ± 8,01 56,27 ± 7,34	0,833	0,411
Preferencia alimentaria				
Educado Sin estudios	24 84	35,95 ± 4,62 35,30 ± 5,39	0,584	0,563

* Prueba t independiente (t).

En la Tabla 5 se presenta la distribución de la media (Media ± DE) de algunos parámetros sanguíneos de las embarazadas. En la Tabla 5 se muestran los valores medios de los parámetros sanguíneos de las embarazadas. La proporción de embarazadas con niveles de hemoglobina < 11 g/dl fue del 12,9%.

**Tabla 5:** Distribución de los promedios de algunos parámetros sanguíneos de las mujeres embarazadas – Kirsehir, Turquía, 2024.

Medias de los parámetros sanguíneos	
Hemoglobina (Media ± DE)	12,35 ± 1,32 (mín. 6,80-máx. 15,40) <11 g/dl = 12,9%.
Hematocrito (Media ± DE)	36,81 ± 3,34 (mín. 24,70-máx. 45,50)
Plaquetas (Media ± DE)	249,18 ± 61,23 (mín. 74-máx. 459)
Proteína (Media ± DE)	66,93 ± 5,33 (mín. 55-máx. 86)
Ácido fólico (Media ± DE)	14,62 ± 5,21 (mín. 3-máx. >20)
Calcio (Media ± DE)	8,85 ± 0,62 (mín. 6,60-máx. 9,90)
Magnesio (Media ± DE)	2,06 ± 0,47 (mín. 1,10-máx. 3,70)
Fósforo (Media ± DE)	3,28 ± 0,84 (mín. 1,60-máx. 9,10)
Hierro (Media ± DE)	64,12 ± 35,98 (mín. 6,00-máx. 171,00)
Zinc (Media ± DE)	82,09 ± 16,60 (mín. 46,32máx. 150,00)
ALT (Media ± DE)	12,74 ± 6,63 (mín. 4,00-máx. 46,00)
AST (Media ± DE)	16,14 ± 5,56 (mín. 8,00-máx. 46,00)
HDL (Media ± DE)	60,59 ± 14,33 (mín. 34,00-máx. 105,00)
LDL (Media ± DE)	114,09 ± 40,96 (mín. 10,50-máx. 212,00)
Glucosa (Media ± DE)	88,22 ± 13,75 (mín. 65,00-máx. 134,00)
Triglicéridos (Media ± DE)	201,60 ± 90,20 (mín. 57,00-máx. 500,00)
TSH (Media ± DE)	2,08 ± 2,34 (mín. 0,01-máx. 22,50)
T4 (Media ± DE)	0,98 ± 0,23 (mín. 0,05-máx. 1,47)
Urea (Media ± DE)	14,88± 3,91 (mín. 6,00-máx. 29,10)
Creatinina (Media ± DE)	0,52 ± 0,08 (mín. 0,34-máx. 0,77)
Albúmina (Media ± DE)	3,84 ± 0,48 (mín. 2,40-máx. 5,17)

## DISCUSIÓN

La edad media de las participantes en este estudio fue superior a la indicada por Irge et al.^(7)^ e inferior a la edad media indicada por Oymak^(3)^, mientras que fue similar a los resultados de Taraz^(10)^. Del mismo modo, los resultados del IMC indicaron diferencias en la distribución de las categorías de IMC en comparación con los de estudios anteriores. Las proporciones de mujeres embarazadas con peso insuficiente, peso normal, pre-obesidad y obesidad en este estudio difirieron de las reportadas por Irge et al.^(7)^. Además, la prevalencia de obesidad leve fue mayor. Estas diferencias pueden atribuirse a variaciones en los hábitos alimentarios específicos de las regiones en las que se realizaron los estudios. Además, un pequeño porcentaje del grupo obeso se clasificó como obesidad mórbida.

Es importante señalar que la obesidad materna puede provocar numerosas complicaciones durante el embarazo y el posparto y aumentar el riesgo de morbilidad neonatal^(19)^. La alta prevalencia de obesidad y sus riesgos asociados en este estudio pone de relieve la necesidad de abordar la obesidad materna y su posible impacto en la salud materna y neonatal.

En comparación con estudios anteriores, este estudio mostró diferencias notables en los niveles educativos y la situación laboral de las mujeres embarazadas. La proporción de mujeres embarazadas con niveles educativos más bajos, como graduadas de la escuela primaria o analfabetas, fue significativamente menor en este estudio que en el estudio de Irge et al.^(7)^. En cambio, la proporción de mujeres embarazadas con educación universitaria o superior fue menor, mientras que la proporción de personas con educación secundaria o inferior fue mayor, en comparación con los resultados de Oymak^(3)^. Estas variaciones pueden atribuirse a las diferencias regionales en el nivel educativo en las distintas zonas de Turquía.

Además, el porcentaje de mujeres embarazadas que eran amas de casa fue inferior al indicado por Irge et al.^(7)^ y Akaç^(11)^. Este hallazgo sugiere un posible cambio en la situación laboral de las mujeres embarazadas a lo largo del tiempo, que puede haber estado influenciado por cambios sociales y económicos. En cuanto a las disparidades de ingresos, el presente estudio informó de una mayor proporción de mujeres embarazadas con unos ingresos familiares que se ajustan a sus gastos en comparación con los resultados de Tandoğan et al.^(20)^. Estas discrepancias en los niveles de ingresos ponen de relieve el impacto potencial de los factores socioeconómicos en la población embarazada y cómo estos factores pueden variar entre las diferentes regiones. En general, la comparación con estudios anteriores reveló variaciones significativas en los niveles educativos, la situación laboral y los niveles de ingresos de las mujeres embarazadas, lo que puede reflejar diferencias regionales y temporales. Estos hallazgos subrayan la importancia de considerar los factores socioeconómicos en la investigación sobre salud maternoinfantil y la necesidad de intervenciones específicas para abordar las posibles disparidades en el acceso a la educación y los ingresos.

Una comparación con el estudio de Irge et al.^(7)^ reveló variaciones en las tasas de consumo de tabaco y alcohol durante el embarazo. En el estudio de Irge et al.^(7)^, la tasa de tabaquismo durante el embarazo fue aproximadamente la mitad de la reportada en este estudio, sin casos de consumo de alcohol. Estas diferencias pueden atribuirse a factores culturales que influyen en el tabaquismo y el consumo de alcohol durante el embarazo.

Además, al comparar la ingesta diaria de agua, el estudio encontró un consumo diario medio de agua más bajo entre las mujeres embarazadas en comparación con los resultados de Taraz^(10)^. Estas diferencias ponen de relieve la posible influencia de los factores culturales y regionales en los comportamientos de salud materna y la importancia de tener en cuenta estos factores en la atención prenatal y las intervenciones de salud pública.

El programa de suplementos de hierro del Ministerio de Salud para mujeres embarazadas es un paso importante para reducir el riesgo de anemia ferropénica durante el embarazo^(21)^. En el presente estudio, la tasa de ingesta de suplementos de hierro fue del 71,3 %, con una tasa del 86,1 % entre las mujeres embarazadas con al menos estudios secundarios. Este hallazgo sugiere que un nivel educativo más alto puede tener un impacto positivo en la frecuencia de la suplementación con hierro.

Además, los niveles medios de hemoglobina de las mujeres embarazadas fueron de 12,35 ± 1,32 g/dL, con una prevalencia de anemia del 12,9 %. Estos hallazgos indican que la mayoría de las mujeres embarazadas que toman suplementos de hierro pueden equilibrar esta afección. Teniendo en cuenta que los datos de la Organización Mundial de la Salud^(22,23)^ indican una prevalencia de anemia del 40,2 % entre las mujeres embarazadas en Turquía, se reafirma la importancia de la suplementación con hierro. Sunuwar et al.^(24)^ informaron que la educación nutricional estaba significativamente asociada con la mejora de los niveles de hemoglobina, la ingesta dietética y el conocimiento nutricional de la anemia. En particular, el 67,6 % de las mujeres embarazadas de este estudio recibieron suplementos de ácido fólico, mientras que el 71,3 % recibieron suplementos de hierro. Estas tasas pueden indicar la necesidad de correcciones específicas en la suplementación con ácido fólico. Estos hallazgos proporcionan información importante sobre el uso de suplementos nutricionales y las quejas relacionadas con el embarazo entre las mujeres embarazadas en Turquía.

La detección de la diabetes mellitus gestacional (DMG) ha sido controvertida en debates recientes. Sin embargo, las organizaciones nacionales e internacionales hacen hincapié en que la realización de una prueba oral de tolerancia a la glucosa entre las semanas 24 y 28 de gestación puede reducir las complicaciones asociadas a la DMG y mejorar los resultados perinatales. Según Çakır y Yeşilçiçek Çalık^(25)^, se determinó que la frecuencia de realización de la prueba oral de tolerancia a la glucosa era del 55,7 %. En este estudio, el 45,3 % de las mujeres embarazadas habían superado las 28 semanas de gestación desde su último período menstrual, y el 48,9 % de ellas se habían sometido a una prueba oral de tolerancia a la glucosa, similar a los resultados del estudio de Çakır y y Yeşilçiçek Çalık^(25)^. La baja frecuencia de la prueba oral de tolerancia a la glucosa puede atribuirse al impacto negativo de la mediana y la escasa alfabetización sanitaria.

Durante el embarazo se producen diversas molestias debido a factores físicos, mentales, sociales y anatómicos relacionados con el crecimiento del útero. Según Karaçam y Özçelik^(26)^, el 99,8 % de las mujeres embarazadas en Brasil se quejan durante el embarazo. En este estudio, la tasa de al menos una molestia durante el embarazo fue del 60,2 %. Esta conclusión, diferente de la de Karaçam y Özçelik^(26)^, puede explicarse por el hecho de que solo el 47,2 % de las mujeres embarazadas incluidas estaban en su tercer trimestre. En otro estudio, la queja más común entre las mujeres embarazadas fue la náusea (34,7 %), seguida de los vómitos (10,9 %)^(7)^. En el presente estudio, las tasas de náuseas y vómitos fueron más bajas y más altas, respectivamente.

En la investigación de Önen y Güngör^(27)^, se informó que la puntuación media de nutrición básica fue de 52 ± 5 en mujeres embarazadas en el segundo trimestre y de 52 ± 6 en el tercer trimestre. En el presente estudio, las puntuaciones medias de nutrición básica fueron más altas. Önen y Güngör^(27)^ también informaron de una puntuación media de preferencia alimentaria de 38 ± 5 en su estudio, mientras que la puntuación media de preferencia alimentaria fue menor en el presente estudio. Estas diferencias pueden estar asociadas con los conocimientos de nutrición y la formación académica. Aunque los niveles medios de triglicéridos y colesterol total fueron inferiores a los comunicados por Brizzi et al.^(28)^, fueron similares a los comunicados por Özdemirci et al.^(29)^.

En nuestro estudio, mientras que los niveles de urea fueron bajos, lo que concuerda con los hallazgos de Özdemirci et al.^(29)^, los niveles de proteína total, albúmina, calcio y hematocrito estuvieron dentro de los rangos de referencia normales, en contraste con sus resultados. Se cree que la disminución de los niveles de urea está asociada con un aumento de la tasa de filtración glomerular, particularmente durante el tercer trimestre. Aunque los niveles medios de folato estaban dentro de los rangos de referencia del laboratorio, eran más altos en el estudio realizado por Comba y Mert^(30)^ (4,9 ng/ml). Esta diferencia puede explicarse por el menor tamaño de la muestra en el estudio anterior en comparación con el presente estudio.

## CONCLUSIÓN

Este estudio es el primero en examinar el estado nutricional de las mujeres embarazadas en la provincia de Kırşehir, en Turquía. Las puntuaciones medias de las mujeres embarazadas en el NKLS para adultos fueron superiores a la mitad de la puntuación media de la escala. Aunque algunos de los parámetros sanguíneos estaban dentro de los valores normales, solo el nivel de urea era inferior a lo normal.

Es fundamental proporcionar educación nutricional a las mujeres embarazadas. La educación nutricional debe comenzar durante el período previo a la concepción, y las mujeres embarazadas deben ser supervisadas e informadas regularmente por dietistas y profesionales sanitarios expertos.
